# Pathological Anatomy

**Published:** 1861-07

**Authors:** John H. Packard

**Affiliations:** Philadelphia


### PATHOLOGICAL ANATOMY.

BY JOHN H. PACKARD, M.D., OF PHILADELPHIA.

I.—*Warty Degeneration of the Vocal Chords in a Child.* Reported by DR. MATTHEWS DUNCAN to the Medico-Chirurgical Society of Edinburgh.

When about five and a half years old, this child, a female, had what was considered a severe cold, and from that time her voice gradually lost strength.

When Dr. Duncan saw her, eighteen months afterward, she could talk plainly, but the sounds were feeble and husky. Her respiration, when full or hurried, was accompanied with a good deal of wheezing. A slight degree of injection and blueness of the face was always present, with an extraordinary expression of anxiety. Her cough was whistling and without expectoration; she was liable to sudden attacks of violent dyspnœa. In one of these attacks she died, having been affected with bronchitis also for about a week previously.

At the autopsy, "the site of the glottis was found occupied by a warty mass, having some papilliform projections, and especially on the left side a distinct polypus. The chink of the glottis was blocked up by tenacious mucus, and was itself extremely small, being a narrow fissure not exceeding two lines in length. The warty mass seemed to spring from the vocal chords, and to have grown upward in flap-like projections, having no connection with the adjacent sides of the larynx. Under the microscope, nothing but large, flat, nucleated cells could be observed in the diseased parts."—(*Edinburgh Medical Journal*, April, 1861.)

II.—*Mediastinal and Pulmonary Cancer.* By DR. J. WARBURTON BEGBIE.

A large, powerful man, fifty years of age, a quarryman, had been in good health until about three weeks before he came under Dr. Begbie's care. Without any exposure to cold, he began at the time mentioned to be affected with a short, dry cough; soon afterward he noticed that his breathing became hurried and panting, especially at the close of a day's work, when he ascended a steep incline. In a few days, the cough and breathlessness obliged him to give up work. His countenance was anxious, fuller on the right side than on the left; this fullness affected the right side of the neck and upper part of the chest. The cough was barking and imperfectly formed; the signs on auscultation and percussion most marked toward the upper part of the right side of the chest, and indicative of some consolidation of the lung tissue. The pupils were alike, and the sight good; there was some headache and giddiness. Hydrothorax and œdema of the right upper extremity became very clearly developed in the course of about two months. Paracentesis was performed, and repeated at intervals of two or three days, until it had been done ten times, more than 550 ounces of fluid having been removed in all. About two weeks before he died, cerebral disorder was manifested; his mind wandered; he had sometimes double vision; his sleep was broken, and he had momentary attacks of unconsciousness. The immediate cause of his death was exhaustion.

An autopsy was made by Dr. Haldane. "On removing the sternum a large mass of encephaloid cancer was disclosed lying under the upper third of that bone, stretching into the right lung, nearly the entire upper lobe of which was involved, while the bronchus was greatly narrowed; the lower lobe of the right lung was much compressed. Serous fluid to the extent of several ounces occupied the right pleural cavity, and at several places the costal and pulmonic surfaces were united by pretty firm adhesions. Some old tubercle was found in both lungs. The mediastinal cancer surrounded the vena cava and large thoracic veins; on the right side it extended downward to the immediate vicinity of the heart. The other organs of the body were healthy; microscopic examination revealed distinct cancer-cells, particularly in the milky juice yielded on pressure from the mass in the lung."—(*Archives of Medicine*, No. 7.)

[This case presents some interesting features, but the statement that "old tubercle was found in both lungs," if correct, embodies the most important point in the account. A powerful, robust man, fifty years old, had "uniformly enjoyed good health, and never suffered from any serious disease" until the onset of a cancerous affection of the mediastinum, probably primarily of the bronchial glands. When had the "old tubercle" been developed? What was its position in relation to the cancerous deposit? Surely the existence of tubercle and cancer, side by side, the organs generally being healthy, *if positively certain*, deserved more than a mere passing mention.—P.]

III.—*New Formation of Lymphatics in the Connective Tissue of the Pleura and Lungs, in a Case of Puerperal Fever.* By M. E. WAGNER, Professor at Leipzig.

At an autopsy on a woman, aged twenty-two years, who had died on the sixth day after delivery, there were found beneath the right pulmonary pleura numerous grayish cords, extending a distance of nearly 2 inches (54 millimètres) from the edge of the lung. The largest of these ramifying cords were about 1 millimètre ($\frac{1}{30}$ inch) in thickness; some were of uniform calibre, others nodose.

The author at first took these cords to be distended lymphatic vessels; but on examining the parts when hardened in alcohol, he saw that these supposed vessels were destitute of proper walls, and constituted canals bounded merely by fibres of connective tissue. The greater number of them were very short (a few millimètres only in length) and ended abruptly. Their contents consisted of granular cells about as large as colorless blood-corpuscles, of a small number of nuclei, and of some albuminous molecules.

The author would trace these newly-formed canals to the corpuscles of the connective tissue, and particularly to those of that lying beneath the pleura; and thinks that this observation goes to prove the origin of the lymphatics from the cells of connective tissue and their prolongations.—(*Gazette Médicale de Paris*, Dec. 15, 1860; from *Archiv für Physiologische Heilkunde.*)

IV.—*Anomaly of the Heart in an Infant.* By M. HERVIEUX.

M. Hervieux exhibited this specimen to the Société Médicale des Hôpitaux, in Paris. It was taken from a child three months old, admitted into the Hospice des Infants Assistés about a month previously. At that time it was in a state of almost complete asphyxia, but recovered so as to take the breast again. Three other attacks of dyspnœa ensued, and on the thirtieth of March the child was seized with complete suffocation, accompanied with cyanosis, loss of temperature, in short, with all the signs of asphyxia; the most vigorous treatment was unavailing, and death took place in twenty-four hours. On opening the thorax, the thymus body was found still very large, so as to cover the heart, which was itself twice the size of the child's fist. The heart being laid open, an abnormal communication was found between its auricles, and a complete absence of the septum between the ventricles, so that there was in fact but one ventricle; there was likewise but one artery, the aorta, the pulmonary artery being deficient. When this artery is wanting, the pulmonary circulation is commonly effected in one of three ways: either by a rudimentary pulmonary artery arising from the aorta, by the persistence of the ductus arteriosus, or finally by means of the bronchial arteries. In the present case, there is neither a rudimentary pulmonary artery nor a persistent ductus arteriosus; and we must admit, although it cannot be demonstrated by an injection, that the bronchial arteries have carried on the circulation.

M. Roger had sometimes, in cases of abnormal communication between the cavities of the heart, perceived a bellows-murmur over the base of the heart during life, so that he could diagnose the lesion which was subsequently proved to exist by post-mortem examination. M. Hervieux had detected no such sound in the present case.—(*Gazette Hebdomadaire*, April 12, 1861.)

V.—*Disease of Aortic Valves Proving Fa'al; Enormous Ascites from an Unusual Cause.* Under the care of DR. D. J. CORRIGAN.

Thomas Hewson, aged thirty-seven, a dock laborer, was admitted into the Whitworth Hospital, on November 22, 1860; he was almost moribund, his face livid and slightly jaundiced, his breathing greatly oppressed, with incessant cough and viscid expectoration. The whole body was anasarcous; the abdomen enormously distended with fluid, very tense, but free from tenderness on pressure. Percussion over the heart was abnormally dull, as was also that over the lower portion of the lungs. The heart's impulse was increased in extent and force, and a double *bruit de soufflet* could be heard especially over its base. Crepitant *râles* existed in the lower part of each lung. The pulse was rapid, and had the rolling character often observed in patency of the aortic valves.

He had been attacked three years before, while at work, with shivering, dyspnœa, cough, and dropsical swelling of the feet and legs; was in the hospital, but recovered sufficiently to go back to work. A year later he had an attack of what would seem to have been peritonitis; this also passed off entirely. About a year after that he noticed an enlargement of his abdomen, and was twice tapped for it at Guy's Hospital, with temporary relief. When admitted into the Whitworth Hospital, he had been suffering for six months from dyspnœa and ascites. His life had been, for nearly twenty years, one of hardship, exertion, and intemperance.

The diagnosis made out was aortic insufficiency, hypertrophy of the heart, congestion, and œdema of the lungs; the ascites could not be accounted for. No treatment was of any avail; he sank and died in a few days.

At the autopsy, "the heart was enormously hypertrophied, weighing twenty-six ounces. The mitral valves were slightly thickened; the aortic were greatly puckered, and quite inadequate to closing the aortic orifice; one of them was perforated, the orifice being ragged and warty. The aorta was diseased, especially in the portion nearest the heart, for the extent of an inch or so. In this situation some calcareous deposits could be felt. The lungs were congested; sections from the lower portions sank in water; and numerous apoplectic clots throughout them explained the immediate cause of death. On laying open the abdominal cavity, and permitting the inclosed fluid to drain off, a most remarkable condition of the parts contained came into view. The whole mass of abdominal viscera were bound together and to the walls of the cavity by extraordinarily developed false membranes. These passed in all directions, varying for the most part from one-sixteenth to one-eighth of an inch in thickness, and in some regions in the form of bands equal in dimension to the columnæ carneæ of the left ventricle. The most remarkable of these bound the stomach to the under surface of the diaphragm. The agglutinated mass of abdominal viscera was with great trouble removed from the body, and at its upper portion, with some difficulty, the liver was recognized; it presented a perfectly globular form, and was reduced to less than one-third its normal dimensions. On making a section, the organ was found perfectly healthy as to structure, but compressed, by a dense false membrane in which it was inclosed, to such a degree as to impede almost completely the transit of blood through it.

"The history of the case, already detailed, corresponded accurately with the post-mortem appearances. The first attack was probably aortitis; and although recovered from, laid the foundation of the cardiac disease, which ultimately proved fatal. The second illness was peritonitis, and the contraction of lymph effused on the surface of the liver, gradually advancing, was the cause of the ascites; the hepatic circulation being obstructed by *pressure from without*, in no less degree than we are accustomed to find it in cirrhosis, by contraction of the capsule of Glisson or *pressure from within*."—(*British Medical Journal*, March 9, 1861.)

### VI.—*Emboli of the Pulmonary Artery.* By MM. TROUSSEAU and DUMONTPELLIER.

MM. Trousseau and Dumontpellier have arrived at the following conclusions as the result of a lengthened series of researches on the subject of emboli of the pulmonary artery:—

1. Fibrinous obstructions of the pulmonary artery are lesions of very common occurrence.

2. Spontaneous coagulations in the pulmonary artery, formed *in situ*, may generally be distinguished from migratory clots derived from the peripheral venous system.

3. There are thus two varieties of pulmonary coagulations—one *primary*, due to some disease of the lung, such as pneumonia, œdema, apoplexy, etc.; and the other *secondary*, resulting from the migration of a peripheral venous clot.

4. Every condition of the system which causes an increased amount of fibrin in the blood, is favorable to coagulation.

5. Local causes of a mechanical nature may act as determining causes.

6. Organic diseases of the heart are most influential in inducing obstructions of the pulmonary artery, partly by the general cachectic condition of the system which they induce, and partly by the mechanical obstacle which they offer to the pulmonary circulation. (Quoted from *L'Union Médicale*, Dec. 13, 1860.)

These conclusions are opposed to the opinions of Virchow, who would make all coagulations in the pulmonary artery depend upon the impaction of a clot detached from vegetations on the lining membrane of the right side of the heart, or from some part of the peripheral venous system. In the *Gazette Médicale de Paris* for Jan. 5, 1861, a case of obstruction of the pulmonary artery is recorded, where there were also contraction with incompetence of the mitral valve, and likewise pneumonia, pulmonary apoplexy, and œdema. Here there was every reason to believe that the obstruction was not due to the impaction of a plug. The obstruction to the circulation through the left side of the heart led to the formation of a large fibrinous clot in the left auricle, which was found adherent after death. This offered an additional obstacle to the onward progress of the blood, the result of which was, first, œdema of the lungs, and then pulmonary apoplexy. This extravasation of blood completely arrested the capillary circulation, and caused a stagnation, first in the smaller, and then in the larger arterial twigs; the clots gradually assuming a fibrinous character. Such appeared the most probable interpretation of the phenomena of the case.—(*British Medical Journal*, Feb. 23, 1861.)

VII.—*Syphilitic Disease of the Liver.* By FRERICHS. (Klinik der Leberkrankheiten, vol. ii.)

According to Frerichs, the syphilitic process manifests itself in the liver in three different forms: 1. As simple interstitial hepatitis and peri-hepatitis. 2. As gummy hepatitis (*gummöse hepatitis.*) 3. As waxy, amyloid, or lardaceous degeneration. All three forms may be found in the same liver, or may exist independently. The last of the three forms is also produced by other cachectic conditions of the system, such as the tubercular diathesis, and the cachexia induced by intermittent fever; and is pretty generally recognized. The first two are less known.

In the bodies of persons who have suffered from constitutional syphilis, white cicatrix-like depressions, of a folded or radiated form, are often found upon the outer surface of the liver, the capsule of which is usually at the same time much thickened, and firmly adherent to the surrounding parts. Sometimes there is only a single depression; at other times, the depressions are so numerous as to give the liver an irregularly lobulated appearance. On careful examination, these depressions are found to be formed by fibrous tissue extending from the thickened capsule more or less deeply into the interior of the gland, the secreting tissue of which is atrophied. The fibrous tissue, in most cases, is dense and tendinous, and contains but few blood-vessels. The larger branches of the portal vein, bile-ducts, and hepatic veins, as a rule, remain uninvolved, except in very rare cases, where the cicatrixes extend deeply into the interior of the gland. Hence this lesion is rarely the cause of either ascites or jaundice.

In the second form, the tissue of the cicatrixes just described contains whitish or yellowish nodules, of a rounded form and dried appearance, usually varying in size from that of a linseed to that of a bean, but occasionally as large as a walnut. Under the microscope, these nodules are found to consist of oil-globules, granular matter, cells loaded with oil, and areolar tissue. They thus resemble in their structural characters the gummy nodules (*Gummiknoten*) which are met with in the subcutaneous areolar tissue, beneath the periosteum, in the testicle, and in other localities, in cases of constitutional syphilis.

In both these two forms of disease, the hepatic tissue intervening between the cicatrixes or nodules is either normal in its character, or more commonly in a state of fatty degeneration. In many cases, also, there is a characteristic hypertrophy, resulting from enlargement of the lobules, which compensates for the loss of substance.

The implication of the liver must, in Frerichs's opinion, be classified with the phenomena of the tertiary stage of syphilis.

The symptoms which accompany the first two forms of the disease are usually so obscure that no suspicion is entertained of the liver being diseased, until the *post-mortem* examination. On the other hand, the symptoms of the waxy or amyloid form are so marked that there is rarely much difficulty in its diagnosis. —(*British Medical Journal*, Feb. 23, 1861.)

VIII.—*Rupture of the Common Duct of the Liver; Formation of a Cyst containing Bile; Death on the Fifty-third Day.* By T. M. DRYSDALE, M.D., of Philadelphia.

A boy, aged thirteen, while standing near a wall, was pressed violently against it by the tongue of a hand fire-engine, which struck him in the right hypochondriac region; he was so seriously injured that he had to be carried home. When seen by a physician, eight hours after the accident, he was extremely prostrated, and complained of intense abdominal pain; he had vomited a good deal of blood. His alvine discharges, as well as his urine, were mixed with blood for several days; the former then became very light in color, the latter dark and muddy, with a green tinge. Slight jaundice was noticed on the third day; he vomited his food, and there was increasing swelling, but no pain or tenderness of the abdomen.

When Dr. Drysdale saw the patient, several weeks after the accident, he found him emaciated and deeply jaundiced, with a pulse of 130, feeble and frequent, a cool, dry skin, laborious and hurried respiration; unable to lie down, restless, with an anxious face, and with intense pain in the right hypochondrium on deep inspiration. On percussing the abdomen, which was much distended, and the superficial veins full and prominent, it was found to yield a clear sound above a line running from the lower border of the eighth rib to the umbilicus; below this line the sounds were dull, except over the ascending colon. The chest was dull on percussion at the lower part of the right side, very clear, with puerile respiration, on the left. Some urine drawn off by means of the catheter was found to contain bile, as did also the fluid drawn from the abdominal cavity in the operation of tapping, which was resorted to a few days subsequently.

The relief derived from the tapping was but temporary; the boy sank, and died in two or three weeks.

An autopsy was made, twenty-eight hours after death. "The body was extremely emaciated; the skin mottled, and of a purplish color. Over the chest, abdomen, and various parts of the body, were large ecchymosed patches, which existed during life. The abdomen was enormously distended. On exposing the abdominal cavity, it appeared to be almost entirely occupied by a large cyst, which was in some parts of a greenish color, in others of a light brown. It was feebly adherent, anteriorly and laterally, to the abdominal walls, as high as an inch above the level of the umbilicus. In handling the cyst, its walls proved to be softened and easily torn, permitting the escape of a large quantity of the same kind of fluid as that removed by tapping.

"On tracing its relations, it was found to dip into the pelvis between the bladder and the rectum, being attached to the peritoneal surface of both of these organs by weak adhesions. It filled the abdominal cavity as high as the umbilicus; and on the right side pushed the diaphragm before it, ascended until it reached the nipple, displaced the small intestines, the cæcum, and the transverse colon, which was folded upon itself, and pressed them all to the left side of the vertebral column. The liver was pushed toward the left side, the right lobe downward, bending it upon itself.

"The upward progress of the cyst in the median line was arrested by the root of the mesentery; winding around which, it rose upon the left side, beneath the intestines, as high as the diaphragm, thus forming a tumor of the horseshoe form.

"The transverse mesocolon was spread over the upper part of the cyst, to which it adhered. On the right side the tumor projected into the epigastric region, anteriorly to the liver, which it concealed, except a small portion of the left lobe, which was firmly adherent by its entire upper surface to the diaphragm. The stomach adhered to the liver, and the pyloric orifice was compressed between this organ and the cyst.

"The peritoneum was discolored wherever it touched the cystic walls; but it presented no signs of inflammatory action, except where the cyst adhered to the surrounding parts. The cyst and liver were so intimately adherent that they were removed together from the body for examination.

"The adhesions between the cyst and the liver were broken down until the transverse fissure of the liver was reached, where the cyst became continuous, in part, with the peritoneal surface of this organ. The gall-bladder was found beneath the cyst, filled with bile, and of the normal size.

"A careful dissection showed the tumor to be a continuation of the peritoneal covering, both of the liver and of the ruptured ductus communis choledochus, the rupture occurring three-quarters of an inch below the cystic duct. The ragged end of the duct projected a quarter of an inch into the cyst. The tumor was lined over the greater part of its internal surface with a layer of lymph."

Dr. Drysdale regards the cyst in this case as formed by the escape of bile into the areolar tissue between the two layers of the gastro-hepatic omentum, this portion of the serous membrane undergoing a regular process of development, so as to constitute the cyst-wall. He quotes from Cooper's Surgical Dictionary a case, probably analogous to his own, but in which the patient recovered, so that the diagnosis could not be absolutely verified.—(*American Journal of the Med. Sciences*, April, 1861.)

IX.—*A Case of Ossification of the Muscles.* By WILLIAM SKINNER, M.B., Lond., etc., of Manchester, Eng.

This singular case is that of a boy thirteen years old, the second of eight children of healthy parents. He was quite healthy until about six years ago, when a swelling was noticed at the back of his neck, without any known cause.

"This disappeared in about a week, another being observed behind the right shoulder. Three weeks later the arms gradually became so stiff that for about three months they were firmly fixed, and the boy was obliged to be fed. He, however, somewhat improved in this respect, and for some time past has been able to feed himself, although with much difficulty. From that time to the present various lumps have formed in different parts of his body, these being principally about the spine and chest; and whenever the boy receives a blow it is invariably followed by a similar swelling. Their appearance is preceded by pain, tenderness, and slight feverishness.

"The condition of the boy at the present time is as follows: He stoops somewhat; the shoulders are contracted; there is no motion in the right joint and very little in the left; the arms cannot be extended, and are in a semiflexed position, crossing the abdomen. The chest is narrow, very much flattened on either side at the junction of the ribs with their cartilages, and these somewhat nodulated. There is no movement of the chest with respiration. The abdomen and lower extremities are well developed, the latter straight, without signs of rickets. There is no movement in the spine, and the scapulæ are fixed; the buttocks and lower extremities present nothing abnormal. On manipulation a number of small projections are felt over the ribs. The right pectoral muscles are completely fixed, and converted into a hard, bony substance. The lower edge of the greater can be traced from a nodule below the right nipple, crossing the axilla, forming a hard and sharp ridge, which is continued into the biceps as far as its insertion into the radius; a number of little bony nodules may be felt along its course, and a long irregular splinter of bone behind it. The muscles of the forearm do not seem to be affected. On the left side there is much the same condition of things as on the right, except that the muscles of the forearm are here beginning to be more or less fixed, and there exists a long, sharp, bony ridge from the outer condyle to about two-thirds of the length of the forearm.

"Posteriorly, on the left side of the neck, and apparently in the trapezius immediately after its origin from the occiput, is a projection of hard, bony substance, about the size and shape of a pigeon's egg. Lower down, between the angle of the scapula and the spine, is a large, hard, and irregular mass. In the dorsal region, at about the tenth and eleventh vertebræ, where the trapezius takes its rise, and apparently formed in the angle of this muscle of the left side, is another hard and somewhat movable mass. It is angular in shape, and extends upward on the side of the spine about one and a half inches, and outward and upward, in the lower fold of the muscle, about one inch. Moreover, in tracing the lower edge of this muscle to its attachment to the scapula some hardness is felt, and here and there sharp spiculæ of bone-like matter. On the right side, about two inches below the lower angle of the scapula, is another hard and irregular projection, about the size of a large egg, the last which has made its appearance; apparently it is incorporated in the lower edge of the latissimus dorsi. In the lumbar region, on each side, hard, bony plates occupy both spaces. The buttocks and lower extremities are quite free from disease, excepting one small nodule, about the size of a common nut, situated over the right os calcis, at the insertion of the tendo Achillis."

This boy exhibited no other structural or functional abnormity. He had taken iodide of potassium, but without any beneficial effect.

"Examples of this peculiar disease may be found recorded by Mr. C. Hawkins in the *Medical Gazette*, 1843 and 1844; by Dr. Rogers in vol. xiii. of the *American Journal of the Med. Sciences;* by Dr. Wilkinson in the *Medical Gazette* for 1846; by Dr. Testelin in the *Gazette Médicale de Paris* for 1839. In the museum of the Royal College of Surgeons of England there is a fine specimen of the disease, the description of which is given in the *Pathological Catalogue*, vol. v. p. 138, No. 3367."—(*Med. Times and Gazette*, April 20, 1861.)

X.—*Hypertrophy of the Connective Tissue of Nerves.* By M. DECHAMBRE.

Among the lesions of the nervous system whose true nature has been elucidated by microscopical study, there is one—the hypertrophy of the connective tissue interposed between the nerve structures—which has acquired more and more importance from recent researches. First described by Rokitansky, this lesion has been made the subject of interesting study by a Swiss physician, M. Demme. We have already called the attention of our readers to the observations of Rokitansky, and would simply recall to their minds that this illustrious pathologist showed the existence of hypertrophy of the connective tissue within the brain, spinal marrow, and different cranial and spinal nerves, and that he traced to this lesion certain paraplegias, various forms of acute convulsions, tetanus, and the general paralysis of the insane. M. Demme, in a work entitled "Pathological Anatomy of Tetanus and of certain other Disorders of the Nervous System," has confirmed in a great degree the opinions put forth by Rokitansky, and has extended the list of affections in which functional lesions of the nervous system may be referred to the condition alluded to. M. Demme compares this alteration to that commonly known as *cirrhosis*, and ascribes to it, in particular, hydrophobia, and certain amauroses observed in patients with Bright's disease.

We do not, of course, hold ourselves responsible for all these assertions, notwithstanding our high regard for their author; but we would mention a case which seems confirmatory of M. Demme's views in regard to the amaurosis of albuminuria, and which was reported to the Swedish Medical Society by Professor Malmsten and Dr. Gyllenschjöld. It was that of a man aged sixty-six, who had labored under albuminuria for several years. Having suddenly lost the sight of the right eye, this patient was attacked quite as suddenly, three days later, with marked amblyopia of the left eye. Only very large objects could be discerned, and this state, which was accompanied by no lesion appreciable by means of the ophthalmoscope, persisted until death. At the autopsy the kidneys were found waxy and granular. The retinæ showed none of the lesions which have been from time to time pointed out in the amaurosis of albuminuria; but the optic nerves, at the point where they penetrate the sclerotica, were almost entirely destroyed by a peculiar growth of connective and elastic tissue. This mass, in which the nerve elements were as if choked, had arisen upon the edges of the sclerotica and in the neurilemma, and prolonged itself, in the form of fibroid extremities, between the nerve cylinders. Here was evidently a hypertrophy of the connective tissue, (*Bindegewebs-Wucherung* of the

Germans.) M. Malmsten added that he had met with this lesion in three subjects, who had become amaurotic at an advanced stage of Bright's disease, and whose retinæ, as well as the other membranes of the eye, were free from appreciable change. Aside from ecchymoses and infiltrations of the retinæ, therefore, the lesion of the optic nerve, pointed out by Demme and Malmsten, should claim the attention of physicians, as one of the causes of the amaurosis of albuminuria, the relative importance of which is yet to be determined.

This case will be of interest in connection with those of M. Von Graefe, where hypertrophy of the interstitial areolar tissue of the optic nerve, with sanguineous injection of the papilla, was consecutive to cerebritis and to intracranial tumors.—(*Gazette Hebdomadaire*, April 12, 1861.)

XI.—*A New Theory as to the Nature of Corpora Amylacea.* By Professor MAYER, of Bonn.

Professor Mayer has remarked that these bodies bear a striking resemblance, in their appearance and reaction with iodine, to the small corpuscles which are found in the joints of tape-worms and in the interior of the cystic entozoa; and in the brain of a sheep, which had died of the staggers, he found, both in the neighborhood of the cœnurus and at a distance from it, an immense number of corpuscles, closely resembling the corpora amylacea found in the brain of man and other mammalia. He likewise discovered an innumerable quantity of similar bodies in the brain and spinal cord of a measly pig. He has not yet had an opportunity of examining the human brain in the bodies of persons who have suffered from tape-worm, but he is of opinion that the corpora amylacea are intimately connected with the presence of entozoa in the system, and that in fact those bodies are nothing more nor less than the corpuscles found in the joints of tape-worms and in the interior of the echinococcus and cœnurus.—(*British Medical Journal*, April 6, 1861; from *Virchow's Archiv*, band ix, hft. 1 and 2.)

XII.—*Deposits of Nitrate of Silver in the Skin and Glands.* By Dr. SIBBALD, of the Royal Edinburgh Asylum.

Dr. Sibbald exhibited to the Medico-Chirurgical Society of Edinburgh microscopical specimens taken from the liver, choroid plexus, and costal cartilages of a man who eight years since took nitrate of silver in solution, during a period of eighteen months, for the cure of epilepsy. After death all the tissues were found to be more or less filled with pigmentary deposit.—(*Edinburgh Medical Journal*, April, 1861.)

